# A Narrative Review of Defensive Medical Practice in Psychiatry

**DOI:** 10.1192/bjo.2022.198

**Published:** 2022-06-20

**Authors:** Khalil Hassanally

**Affiliations:** CNWL, London, United Kingdom. University of Bristol, Bristol, United Kingdom

## Abstract

**Aims:**

Defensive medical practice has become an increasingly global phenomenon and encompasses all medical specialties. In the UK it was defined in the case of *Sidaway v Board of Governors of the Bethlem Royal Hospital* [1985] UKHL 1 (21 February 1985) as “the practice of doctors advising and undertaking the treatment which they think is legally safe even though they may believe that it is not the best for their patient”. This narrative review surveys the literature to establish the forms in which defensive practice may manifest itself within psychiatry.

**Methods:**

In this narrative review, various terms pertaining to defensive medical practice in psychiatry were searched in both medical and legal databases.

**Results:**

Though the literature in psychiatry compared to other medical specialties is more limited, some common themes occur across all jurisdictions surveyed. Defensive psychiatric practice included admitting the patient even though they may be managed within the community (as reported by 21% of psychiatrists surveyed in the North of England) and employing more coercive practice, either using the mental health legislation or implied or actual threats. Once hospitalised, defensive practice manifests itself by placing patients on higher levels of nursing observations than necessary.

Across inpatient and outpatient settings between one and two thirds of psychiatrists reported altering the way they document to attend to medicolegal considerations. Prescribing habits were also altered due to fears of litigation; an Israeli study found that almost half of psychiatrists surveyed reported they prescribed smaller doses of medication than what they felt was required to pregnant woman and ninety percent reported the same when it came to the treatment of elderly patients.

When looked at by seniority it was felt that junior doctors were more prone to admitting patients defensively than consultants. In this respect, psychiatry differs from most other medical specialties as, in general, the evidence suggests that increased seniority is more likely to lead to admission.

**Conclusion:**

Defensive practice in psychiatry appears to be widespread and takes a number of different forms. However, the research in psychiatry is limited and does not explore key areas common to other medical specialties such as clinician avoidance of certain cases or increased use of diagnostic tests. Furthermore, there is little examination of how psychiatrists may utilise mental health legislation within their defensive practice.

